# Assessment of Vitamin D-Binding Protein and Early Prediction of Nephropathy in Type 2 Saudi Diabetic Patients

**DOI:** 10.1155/2018/8517929

**Published:** 2018-04-03

**Authors:** Manal S. Fawzy, Baraah T. Abu AlSel

**Affiliations:** ^1^Department of Biochemistry, Faculty of Medicine, Northern Border University, Arar, Saudi Arabia; ^2^Department of Medical Biochemistry, Faculty of Medicine, Suez Canal University, Ismailia, Egypt; ^3^Department of Microbiology, Faculty of Medicine, Northern Border University, Arar, Saudi Arabia

## Abstract

Early detection of diabetic nephropathy (DN) represents a great challenge in an attempt to reduce the burden of chronic kidney diseases in diabetic patients. This study aimed to investigate the potential early prediction role of urinary vitamin D-binding protein (uVDBP) for the diagnosis of DN and to examine the possible correlation to serum VDBP, high-sensitivity C-reactive protein (hs-CRP), and insulin resistance in these patients. Serum and urine samples were obtained from 40 healthy volunteers and 120 patients with type 2 diabetes divided into 3 groups: normoalbuminuria, microalbuminuria, and macroalbuminuria (urinary albumin excretion rate < 30, 30–300, and >300 *μ*g/mg, resp.); *n* = 40/group. Serum and urinary VDBP levels were quantified by ELISA. Insulin resistance has been assessed by homeostasis model assessment index (HOMAI). Correction for urine creatinine concentration was applied for urinary quantitative measurements. uVDBP levels were significantly elevated in micro- and macroalbuminuria patient groups compared with those of the normoalbuminuria patient group and controls (820.4 ± 402.8 and 1458.1 ± 210.0 compared with 193.1 ± 141.0 and 127.7 ± 21.9 ng/mg, resp.) (*P* < 0.001). There was significant correlation between serum and urinary levels of VDBP in total patient group. Receiver operating characteristic analysis of uVDBP levels showed optimum cut-off value of 216.0 ng/mg corresponding to 98.8% sensitivity and 80.0% specificity and an area under the curve of 0.973 to discriminate the normoalbuminuria from the microalbuminuria groups. In multivariate analysis, ordination plot showed obvious demarcation between the study groups caused by the higher levels of uVDBP and albumin/creatinine ratio among other variables. The study findings suggested a possible clinical application of uVDPB as an early and a good marker for the detection of early renal disease in type 2 DM Saudi patients. Large-scale validation studies are warranted to confirm the results before including uVDBP with the available list of other conventional biomarkers.

## 1. Introduction

Diabetes mellitus (DM) is one of the most growing chronic diseases worldwide, and its incidence has been speculated to rise up to 500 million by 2030 [1]. Recently, Saudi Arabia has been enlisted in the top ten countries with high diabetes prevalence (23.9%) due to the major socioeconomic changes that have occurred and associated with major changes in the person's lifestyle [2]. Diabetic nephropathy (DN) is a progressive kidney disease caused by glomerular as well as tubular structural and functional alterations which is induced by glucose homeostasis disturbance [3]. It occurs in approximately 30% of diabetic patients, and it is one of the main causes of end-stage renal disease (ESRD) [4]. Over the past few decades, major advances were achieved in its diagnosis and treatment, albeit it is still representing one of the late complications which associated with increased risk for cardiovascular morbidity and mortality [3]. Measuring of urine albumin levels (albumin-to-creatinine ratio) has been used conventionally to detect DN severity [5–9]. Even though persistent microalbuminuria (30–300 *μ*g/mg) or macroalbuminuria (levels > 300 *μ*g/mg) [10] was considered as the best available, noninvasive marker and predictor for DN risk and its progression to ESRD [3], certain studies have shown it to have inadequate specificity and sensitivity [11–13] and it does not cover all patients with renal impairment [14]. In addition, microalbuminuria was detected in other patients with kidney impairment as in cases of hypertension and glomerular basement membrane dysfunction [15]. Thus, additional studies for novel noninvasive risk markers in body fluids are required, and feasible measures for the diagnosis of DN prior to advanced renal dysfunction are considered to be of clinical importance with a public health implication [16, 17].

Vitamin D-binding protein (VDBP) (primary accession number P02774; http://www.uniprot.org/uniprot/P02774), also known as gc-globulin, is a 58 kDa glycoprotein which serves as the main carrier protein for circulating vitamin D and its metabolites, supporting the bioavailability of active 1,25-dihydroxyvitamin D (1,25(OH)_2_D) and its precursor 25-hydroxyvitamin D (25OHD) [18, 19]. The vital role by which VDBP plays for maintaining the serum levels of the bioactive vitamin D is important for the function of a wide variety of tissues which show development of a number of diseases associated with changes in VDBP activity [20].

In addition to the transport function, VDBP is the parent molecule of VDBP-maf (macrophage-activating factor). This latter molecule is the deglycosylated product form of VDBP and has been reported to be a potent antiangiogenic and antitumorigenic molecule [21]. Moreover, VDBP is important in the actin-scavenger system, participating in the immune responses and the inflammatory processes [20]. Studies concerning the actions of VDBP in the kidney have received increased attention and have reported that VDBP is vital for 1,25(OH)2D biosynthesis within renal proximal tubules, in which it binds 25OHD and the complex is actively recovered from the glomerular filtrate through megalin-mediated receptor endocytosis [22, 23]. Clinically, it has been demonstrated that exaggerated excretion of urinary VDBP is associated with tubular dysfunction [24]. Therefore, it was hypothesized that the loss of urinary VDBP is likely to be elevated in diabetic patients and particularly accentuated in those patients with DN [25].

In addition, it has been demonstrated that the presence of vitamin D deficiency or insufficiency in patients with diabetes is independently associated with the development of DN. Moreover, exaggerated urinary excretion of VDBP was observed in patients with type 1 diabetes, which contributed mechanistically to vitamin D deficiency in this disease [24, 26–28].

Therefore, a further possibility for the potential elevation of urinary VDBP levels identified in some studies may be associated with the relatively lower serum level and vitamin D levels. Hence, further studies are required to clarify the role of VDBP in the pathogenesis of DN in particularly type 2. This study was designed to evaluate the urinary vitamin D-binding protein in patients with type 2 DM presented with different degrees of DN and to examine the possible correlation to the available clinico-laboratory parameters to explore its validity as an early, specific, and sensitive biomarker for nephropathy and its severity in Saudi diabetic patients in the northern area of the KSA. This is a preliminary step in an attempt to early implement the preventive measurements and control the occurrence of renal failure in these patients.

## 2. Subjects and Methods

### 2.1. Participants

One hundred and twenty diabetic patients (DM type 2) and 40 age- and sex-matched apparently healthy controls have been enrolled in the current preliminary case-control study. Early morning serum and urine samples have been obtained on the same day from all participants. Patients who were attending the Prince Hospital Outpatient Diabetic Clinics, Northern Borders Area, Saudi Arabia, were divided into 3 patient groups: (1) normoalbuminuria group (urinary albumin excretion rate < 30 *μ*g/mg), (2) microalbuminuria group (at least two of three consecutive urine samples with albumin excretion rate 30–300 *μ*g/mg), and (3) macroalbuminuria group (albumin excretion rate > 300 *μ*g/mg) [10], (*n* = 40 per group). Patients with active urinary tract infection, renal disease other than diabetic nephropathy (diagnosed by renal biopsy according to recommended protocols adopted from international standards for diagnosis of nondiabetic renal disease) [29], neoplastic disorders, severe liver disease, active or chronic infection or inflammatory disorders, hematological diseases, pregnancy or a recent history of acute myocardial infarction, stroke, or occlusive peripheral vascular disease have been excluded. Patients' medical data have been reviewed from their medical records. In addition, blood pressure, body weight, and body mass index have been measured. The control subjects were randomly selected from the general population. They had no signs or clinical symptoms of chronic diseases or cancer, and they did not take any regular medication. The study was conducted in accordance with the ethical standards of the institutional and national research committee and with the Helsinki Declaration and its later amendments or comparable ethical standards. It was approved by the Medical and Bioethics Local Committee of Northern Borders University. All participants provided written informed consent to participate in the study after being informed with its purpose.

### 2.2. Biochemical Analysis

Venous blood samples (8 ml) were withdrawn after an overnight fast (10–12 h); 7 ml was collected on a plain tube for serum separation after centrifugation at 2500 rpm × 15 minutes, then stored frozen at 80°C until the time of laboratory analysis. The remaining 1 ml from the blood sample was placed in EDTA tube for glycated hemoglobin (HbA1c) determination (Cobas Integra, Roche Diagnostics, USA).

Routine laboratory measurements including blood urea nitrogen (BUN), serum creatinine, fasting blood sugar (FBS), and lipid profile (total cholesterol (TC), high-density lipoprotein cholesterol (HDL-c), and triacylglycerol (TG)) were done using commercially available kits on Cobas Integra 400 plus Biochemical analyzer (Roche Diagnostics). Low-density lipoprotein cholesterol (LDL-c) concentration was calculated by the Friedewald equation [30].

A clean-catch midstream urine samples (nearly 20 ml) were collected into a sterile plastic tube and then centrifuged for 10 minutes at 3000 rpm, 4°C, to remove cell debris and particulate matter. The supernatant was stored at −80°C for further analysis. Repeated freeze-thaw cycles were avoided. Spot urinary albumin and creatinine concentrations were measured (Siemens Healthcare Diagnostics Inc., USA) and expressed as the urinary albumin (*μ*g)/creatinine (mg) ratio (UACR). Glomerular filtration rate was estimated (eGFR) using the four variable Modification of Diet in Renal Disease GFR formulas (age, sex, race, and serum creatinine) which are as follows: eGFR = 186 × (serum creatinine^–1.154^) × (age^–0.203^) × (0.742 if female) [31]. Quantitative estimation of serum hypersensitive C-reactive protein (hs-CRP) was done by means of particle enhanced immunonephelometry using BN system (Dade Behring, USA).

Hormonal assay for insulin was measured by Electro-chemiluminescence Immunoassay (Cobas, Roche Diagnostics, USA). Insulin resistance was assessed using HOMA model (homeostasis model assessment index) = fasting insulin (*μ*U/ml) × fasting glucose (mmol/l)/22.5 [32].

Serum and urinary VDPB were measured in duplicate using a Human Vitamin DBP Quantikine ELISA kit (DVDBP0; R&D Systems, Minneapolis, MN, USA). The assay was performed according to the instructions recommended by the manufacturer. The standard curve was created using the lyophilized human VDBP standard preparation supplied with the assay. Following the colorimetric reaction, the optical density (OD) readings were converted to concentrations in ng/ml based on quantification of the OD at 450 nm. The levels of uVDBP were normalized according to urine Cr concentrations (to avoid the influence of urine volume) and presented as uVDBP : Cr ratio (ng/mg of Cr) [33]. The intra-assay coefficient of variations of the urinary and serum VDBP were 6.2 and 5.5%, respectively.

### 2.3. Statistical Analysis

Statistical analysis was performed using SPSS, version 22 (Statistical Package for the Social Sciences, SPSS Inc., Chicago, Illinois, USA). Normally distributed continuous values were expressed as means ± SD and compared using analysis of variance (ANOVA) test followed by Tukey HSD post hoc test for multiple comparisons, whereas nonnormally distributed values were expressed as median with interquartile range (IQR) and compared by the Kruskal Wallis test followed by Tukey HSD multiple comparison test. Categorical variables were presented as percentage and compared by chi square test. Moreover, Pearson's correlation coefficient was used to test correlations between serum and urinary VDBP and other variables. Linear regression analysis was applied to allow us to estimate the association between a given independent variable and the outcome holding all other variables constant and to provide a way of adjusting for potentially confounding variables that have been included in the model. Receiver operating characteristics (ROC) analysis was used to calculate the area under the curve (AUC) for uVDBP and to find the best cut-off values to identify diabetic nephropathy. The results with *P* < 0.05 were considered statistically significant. Finally, for clustering the study participants according to clinico-laboratory variable interaction, PC-ORD version 6.0 was employed to run the multivariate analysis. Bray-Curtis ordination and two-way hierarchical cluster analysis were run to identify the combination of variables that could discriminate between the patient and control groups. Data profile was first examined. No outliers were detected, and no transformation was required. Ordination was run to visualize data of participants along the axis according to their resemblance. Sorensen coefficient, Euclidean residual distance, and variance-regression endpoint selection method were adjusted to calculate scores for factors by weighted averaging. In cluster analysis, flexible beta linkage method at −0.75 and Sorensen distance measure were selected [34].

## 3. Results

### 3.1. Baseline Characteristics of the Study Groups

Clinical and biochemical data for the diabetic patients categorized according to the level of albuminuria compared to the control group are shown in Tables 1 and 2, respectively. Patients and controls were matched with age and gender. No significant differences were observed with regard to history of smoking or presence of hypertension. However, diabetic patients showed a higher body mass index and a higher frequency of positive family history for diabetes. There was a significant increase of serum TG in the macroalbuminuria group compared to other groups (*P* < 0.001). For insulin resistance expressed as HOMA model, it was significantly higher in the diabetic groups versus the control group (*P* < 0.001) as expected.

### 3.2. Serum and Urinary VDBP Profiles

**Figure 1:**
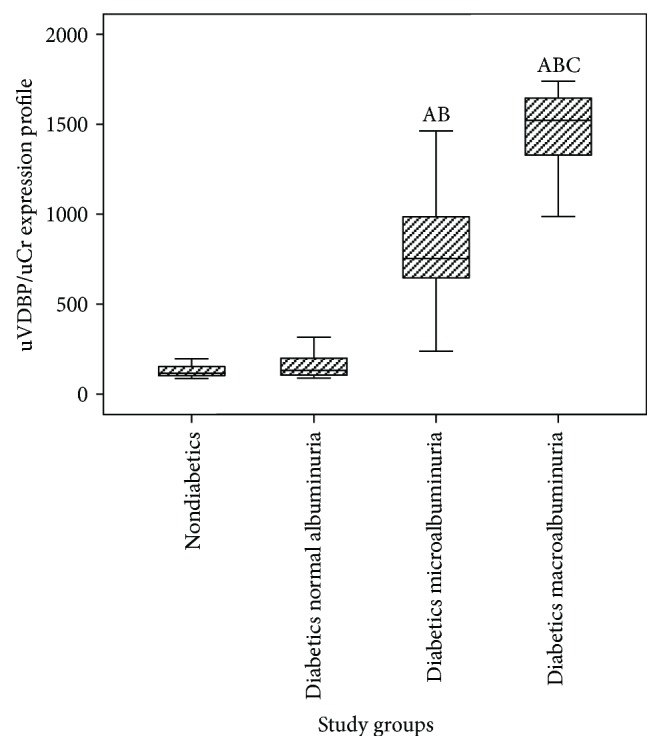
Urinary vitamin D-binding protein levels among the study groups. uVDBP, urinary vitamin D-binding protein; uCr, urinary creatinine. ^A^Compared to the control group. ^B^Compared to the diabetic normal albuminuria group. ^C^Compared to the diabetic microalbuminuria group. Kruskal-Wallis test and Tukey HSD multiple comparison test were applied.

**Figure 2:**
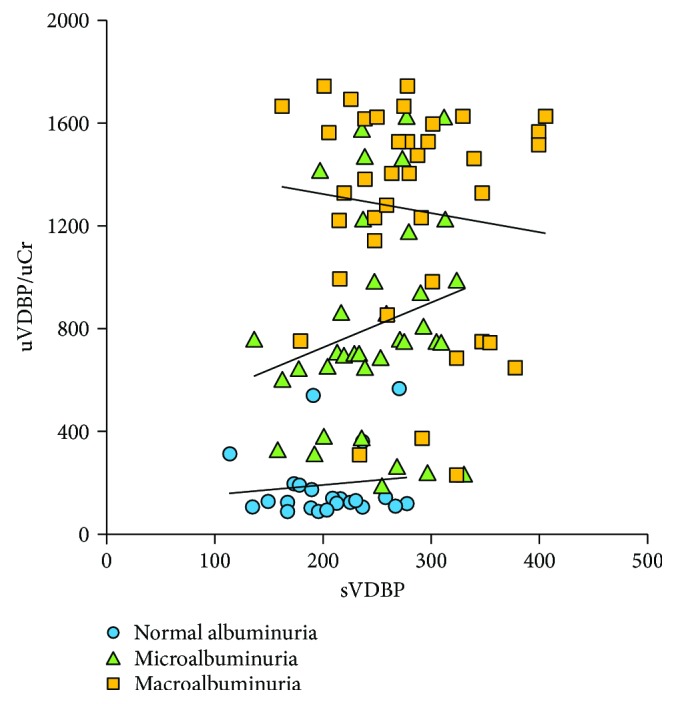
Correlation between urinary VDBP/Cr and serum VDBP levels in patient subgroups.

Mean urinary VDPB levels were significantly different among the study groups (*P* < 0.001) with increasing levels with the degree of albuminuria (Table 2 and Figure 1). uVDBP levels were significantly elevated in the micro- and macroalbuminuria patient groups compared to the normoalbuminuria diabetic group and normal controls (820.4 ± 402.8 and 1458.1 ± 210.0 compared with 193.1 ± 141.0 and 127.7 ± 21.9 ng/mg, resp.) (*P* < 0.001). In addition, the serum VDBP levels were also significantly elevated in the patient subgroups: normo-, micro-, and macroalbuminuria compared to controls (Table 2). There was a significant positive correlation between serum and uVDBP levels in the whole patient group, albeit this significance was not evident when patients were stratified according to the levels of albuminuria (Table 3, Figure 2).

**Figure 3:**
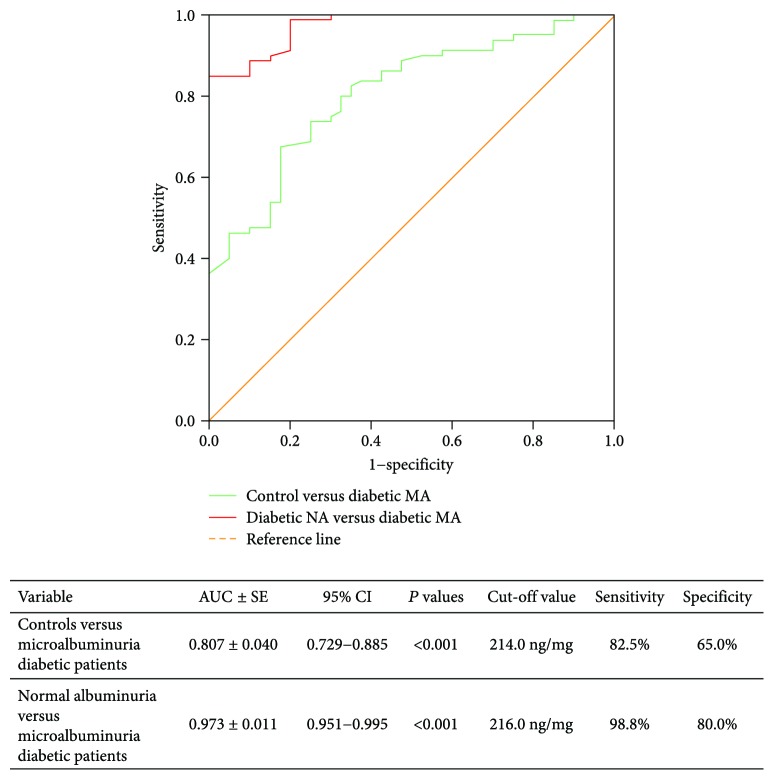
Diagnostic performance of uVDBP to detect patients with DN. NA: normoalbuminuria; MA: microalbuminuria; AUC: area under curve under the nonparametric assumption; 95% CI: 95% confidence interval; SE: standard error.

Receiver operating characteristic analysis of urinary VDBP levels showed optimum cut-off value of 216.0 ng/mg corresponding to 98.8% sensitivity and 80.0% specificity and an area under the curve of 0.973 to discriminate the normoalbuminuria from microalbuminuria groups (Figure 3).

Pearson's correlation between uVDBP levels and the different clinico-laboratory parameters showed its significant correlation with family history of diabetes, HbA1c %, LDL-c, total serum protein levels, and urinary albumin/creatinine ratio (*r* = 0.309, 0.584, −0.244, −0.202, and 0.775, resp.) in the total patient group (Table 3).

Univariate logistic regression analysis was executed to identify which of the predictors (independent variables) are significantly contributing to DN (Table 4). Percent accuracy classification of the model was 94.2%. Serum VDBP and uVDBP levels showed the only statistically significant independent predictors to microalbuminuria (Table 4). While the odds ratios were statistically significant, the values were near 1 (i.e., the magnitudes of the effect were 1.028- and 1.01-fold increase for sVDBP and uVDBP levels, resp.). A larger study is needed to generate a more precise estimate of effect and confirm these associations.

### 3.3. Multivariate Analysis

**Figure 4:**
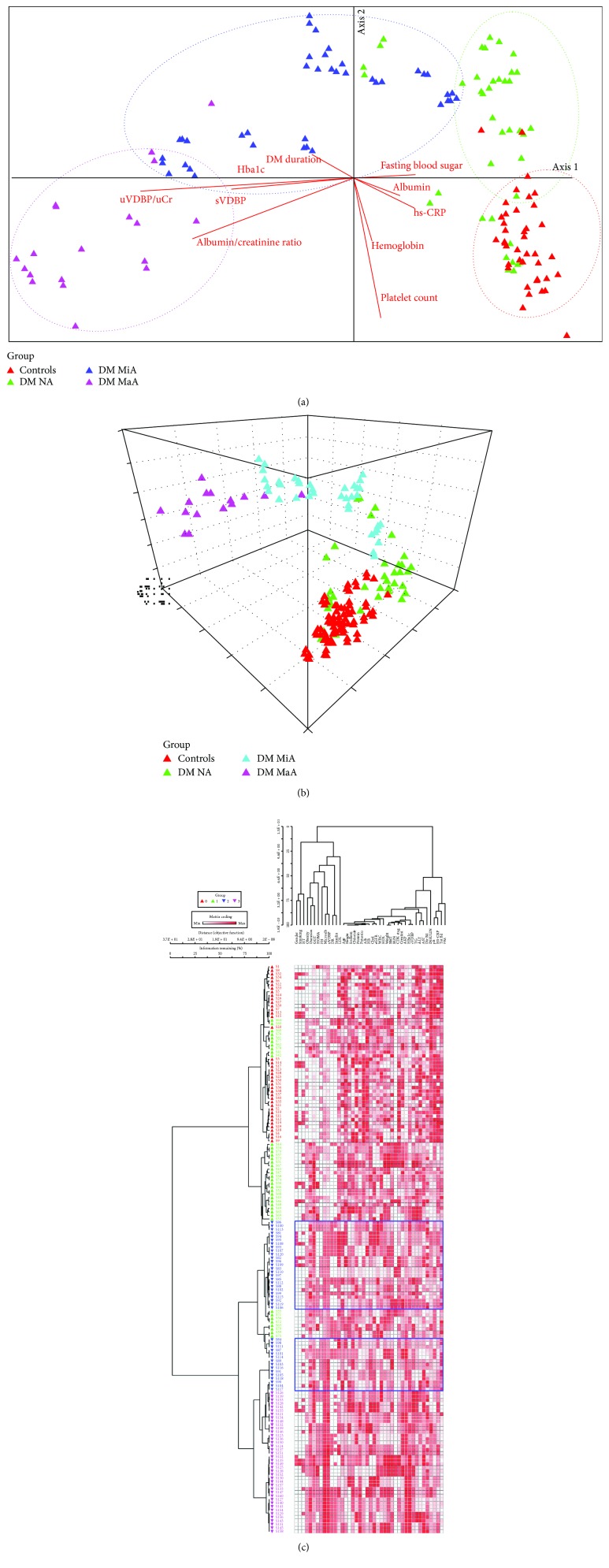
Multivariate analysis of the study participants. (a) Despite some mixing between groups, the study participants were clustered into four distinct groups: (1) one control group (red) which was mostly affected by high hemoglobin level, (2) diabetic without albuminuria (green) at the positive direction of axis 1 which was influenced by high fasting blood sugar levels, (3) diabetic microalbuminuria (blue), and (4) diabetic macroalbuminuria (pink) in the negative direction of axis 1 which was greatly affected by high level of uVDBP/Ucr, sVDBP, albumin/creatinine ratio, and HbA1c. (b) 3D ordination plot shows also separation between the controls (red) and other patient subgroups along axes 1 and 2. uVDBP was a potential factor contributing to this separation. (c) Two-way hierarchical cluster analysis shows clear cut between macroalbuminuria and the other 3 groups with 100% separation. However, the diabetic normoalbuminuria group shares some characteristics with the normal controls and microalbuminuria group.

Ordination plot showed obvious demarcation between the study groups. In axis 1, uVDBP/uCr, sVDBP, and HbA1c were the most effective classifier in the negative direction explaining 96.7%, 55.6%, and 53.9% of axis variability. Whereas, axis 2 was mostly affected by platelet count, hemoglobin, and albuminuria/creatinine ratio with *R*^2^ of 44.8%, 20.3, and 19.3%, respectively. Two-way cluster analysis also demonstrated clustering of patients and controls into separate groups (Figure 4).

## 4. Discussion

The identification of novel biomarkers of the early stages of DN is mandatory in an attempt to reduce the burden of chronic kidney diseases in diabetic patients [3]. To evaluate whether uVDBP levels could be a novel noninvasive biomarker for DN in a sample of Saudi population, the current study results demonstrated that the uVDBP levels were highly elevated in Saudi patients with DN and were correlated significantly with the severity (degree of albuminuria) of DN. Interestingly, the human *VDBP* gene is a member of a multigene cluster [35] residing on chromosome 4 and coding for related albumin proteins (i.e., albumin, alpha-fetoprotein, and afamin) which have structural and functional similarities [36]. In the normal kidney, VDBP as a 25-(OH) vitamin D3/VDBP complex is reabsorbed by megalin-mediated endocytosis and catabolized by epithelial cells of the proximal tubules contributing to the reduction of its urinary excretion levels [37]. Clinically, it has been found that excessive excretion of uVDBP could indicate tubular dysfunction [38–40] which was considered as one of the early hallmarks of DN [41]. In line with our findings, Rao et al. [42] and Nauta et al. [43] reported elevated uVDBP levels among other DN proteomic markers in patients with diabetes compared to nondiabetics, especially when albuminuria is present. Several recent studies, in addition, support the marked increase in the uVDBP excretion in patients with normo-, micro-, and macroalbuminuria in type 1 [24, 44] as well as type 2 [25, 45, 46] diabetes, compared with that in the controls in different populations. Despite the specific mechanisms underlying the increased uVDBP excretion in patients with DN were not fully uncovered, several evidences support that the enhanced excretion of megalin/cubilin (i.e., multiligand endocytic receptors expressed in the brush border of proximal renal tubular cells and participate in the reuptake of filtered low-molecular weight proteins like albumin and VDBP from the glomerular filtrate) in the urine of patients with DN could play a role [47, 48]. Another speculated mechanism could be related to the renal injury which is associated with DN progression [25] independent of the presence of albuminuria. This speculation could be supported by the findings of Mirkovic´ et al. [49] and Chaykovska et al. [50] who found that uVDBP was increased along with the severity of renal damage independently of albuminuria in a rat model of proteinuric nephropathy that responded partially to an intensified renoprotective therapy in the former study and in contrast-induced nephropathy in the latter one. It has been postulated that the damaged tubular epithelial cells in areas of tubulointerstitial fibrosis may no longer be capable to deal with VDBP, resulting in its gross loss into the urine [49]. Additionally, it has been demonstrated that the major factors involved in the development of glomerulosclerosis and interstitial fibrosis of DN (e.g., TGF-*β* and angiotensin II) [51, 52] could negatively regulate the receptor-mediated endocytosis [53, 54], participating in enhanced uVDBP excretion.

In the current study, receiver operating characteristic analysis of uVDBP levels showed an area under the curve of 0.973 to discriminate the normoalbuminuria from microalbuminuria groups. Furthermore, the multivariate analysis has confirmed the role of uVDBP as a putative biomarker that could be used for clear demarcation between diabetic patients and the controls. This could support the rationale for using uVDBP as an emerging biomarker for early prediction and detection of DN as evidenced by other studies [25, 44, 45]. In addition, consistent with the results of these studies, our findings showed uVDBP positively correlated to indices of worsening glycemic control as high HbA1c % and to ACR. This latter correlation with the level of albuminuria was supported by Doorenbos et al. observation in which uVDBP excretion responded to antiproteinuric treatment in their chronic kidney disease patients [55]. Rather than family history of diabetes, LDL-c, and total protein levels, uVDBP did not show significant correlation with other clinical and laboratory features in the study population. This finding was in line with Leong et al.'s [56] results who concluded from their follow-up of 2254 Canadian individuals for ten years that apart vitamin D levels, VDBP has no demonstrable causal effect on any of the investigated cardiometabolic traits in their study.

**Figure 5:**
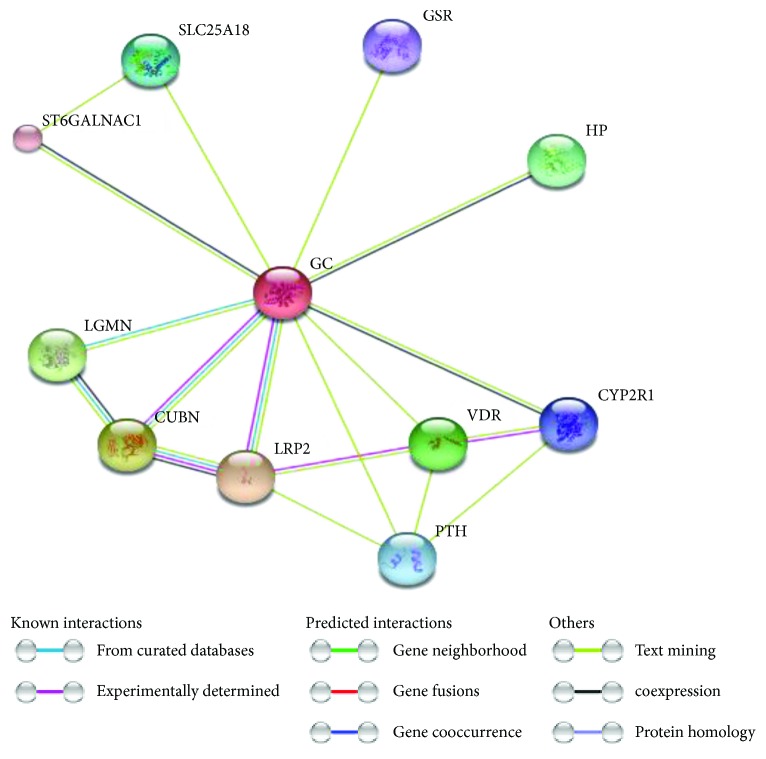
STRING analysis of vitamin D-binding protein (i.e., Gc, group-specific component protein) (accession number P02774) with its predicted functional partners. Network nodes represent proteins; each node represents all the proteins produced by a single, protein-coding gene locus. Edges represent protein-protein associations (i.e., contribute to a shared function; this does not necessarily mean they are physically binding each other). The line colors of the edges indicate the type of interaction evidence that is explained in the figure key (right side). LRP2, low-density lipoprotein receptor-related protein 2 or megalin, is a multiligand endocytic receptor that is expressed in many tissues but primarily in absorptive epithelial tissues such as the kidney; CUBN, cubilin, an intrinsic factor-cobalamin receptor cotransporter which plays a role in lipoprotein, vitamin, and iron metabolism, by facilitating their uptake. LGMN, legumain, has a strict specificity for hydrolysis of asparaginyl bonds required for normal lysosomal protein degradation in renal proximal tubules and plays a role in the regulation of cell proliferation via its role in EGFR degradation; VDR, vitamin D (1,25-dihydroxyvitamin D3), is a nuclear receptor that mediates the action of vitamin D3 by controlling the expression of hormone-sensitive genes and plays a central role in calcium homeostasis; HP, haptoglobin, captures and combines with free plasma hemoglobin to allow hepatic recycling of heme iron and to prevent kidney damage. SLC25A18, solute carrier family 25 (glutamate carrier), is member 18 which involved in glutamate transport with H^+^ across the inner mitochondrial membrane; PTH, parathyroid hormone; CYP2R1, cytochrome P450, family 2, subfamily R, polypeptide 1 which has a D-25-hydroxylase activity on both forms of vitamin D: D2 and D3. GSR, glutathione reductase, maintains high levels of reduced glutathione in the cytosol. ST6GALNAC1 transfers a sialic acid, N-acetylneuraminic acid (NeuAc), in an alpha-2,6 linkage to O-linked GalNAc residues [data source: https://string-db.org, version 10.5].

Unexpectedly, serum VDBP levels were significantly elevated in patient subgroups relative to the controls, although the urinary loss of VDBP was enhanced. This finding could indicate increased production of serum VDBP as a compensatory mechanism as speculated by Kalousova and coworkers [57]. In addition, VDBP can interact with many functional partners (Figure 5) and have additional vital metabolic and immunological roles apart its involvement in vitamin D transport and storage [20, 58, 59]. For example, as diabetes could be considered as a chronic state of low grade inflammation [60, 61], this could contribute in part to the increasing levels of serum VDBP as a response to the proinflammatory state. Furthermore, previous reports suggested that serum VDBP level differences that are associated with common diseases could support its role either as an intermediate in several biological pathways or as an upstream player affecting vitamin D effects on common diseases. This speculation was supported by analogy to other circulating steroidal hormone transporters, including sex hormone or corticosteroid binding globulins, the major effectors of steroid action independent of their function as carriers [62].

Some limitations of our study include the relatively small sample size, the cross-sectional design, and the fact that the study lack of inclusion of other types of nephropathies to which uVDBP could be assessed to confirm its specificity of this biomarker for DN. Hence, it is highly recommended that uVDBP to be validated in well-characterized larger scale cohorts with longitudinal follow-up and assessment of its response to proper DN management, as well as addressing the associations between uVDBP and the other types of nephropathy in the clinical setting. Furthermore, as it is well known, the genetic variability of VDBP could affect its circulating levels [63]; complicating the interpretation process of the serum VDBP, it is highly recommended considering its genetic variants when to interpret or associate serum VDBP levels with the disease phenotype.

## 5. Conclusions

Taken together, the current results suggested that uVDBP could be implicated in combination with other conventional biomarkers for the early prediction of DN in Saudi population. This could improve the early diagnosis of DN and help in prevention of progress to ESRD by applying early and personalized targeted therapy after validation of the findings in larger scale studies.

## Figures and Tables

**Table 1:** Clinical and demographic characteristics of the study groups.

Variables	Control group (*n* = 40)	Diabetic groups			*P* values
		Normal albuminuria	Microalbuminuria	Macroalbuminuria	
		(*n* = 40)	(*n* = 40)	(*n* = 40)	
Age (years)	47.6 ± 13.6	50.6 ± 7.8	50.4 ± 12.0	45.9 ± 6.5	0.127
Gender, *n* (%)					
Females	32 (80.0)	35 (87.5)	33 (82.5)	34 (85.0)	0.821
Males	8 (20.0)	5 (12.5)	7 (17.5)	6 (15.0)	
Weight (kg)	74.9 ± 14.3	80.1 ± 15.1	77.2 ± 11.7	80.4 ± 18.5	0.315
Height (cm)	166.1 ± 5.2	156.4 ± 7.9^a^	158.3 ± 9.5^a^	154.5 ± 4.4^ab^	**<0.001**
BMI (kg/m^2^)	27.2 ± 5.5	32.8 ± 6.2^a^	30.8 ± 4.1	33.7 ± 7.5^a^	**<0.001**
Obesity, *n* (%)	12 (30.0)	30 (75.0)	18 (45.0)	25 (62.5)	**<0.001**
Hypertension, *n* (%)	14 (35.0)	11 (27.5)	9 (22.5)	10 (25.0)	0.625
Smoking, *n* (%)	7 (17.5)	2 (5.0)	3 (7.5)	3 (7.5)	0.227
FH of DM	11 (27.5)	17 (42.5)	24 (60.0)	28 (70.0)	**0.001**
DM duration (years)	—	5.5 ± 2.5	6.1 ± 2.7	6.8 ± 2.1	0.063
Insulin therapy, *n* (%)	—	17 (42.5)	19 (47.5)	20 (50.0)	0.791
DR, *n* (%)	—	14 (35.0)	16 (40.0)	19 (47.5)	0.519
CKD, *n* (%)					
Stage 1	—	34 (85.0)	34 (85.0)	30 (75.0)	**0.013**
Stage 2	—	6 (15.0)	6 (15.0)	10 (25.0)	

Data are expressed as mean ± SD or *n* (%). DM: diabetes mellitus; BMI: body mass index; FH: family history; DR: diabetic retinopathy; CKD: chronic kidney disease. Chi square test was used for qualitative variables. One-way ANOVA for quantitative variables followed by Tukey HSD post hoc test for multiple comparisons. ^a^Compared to the control group; ^b^compared to microalbuminuria. Bold values indicate significance at *P* < 0.05.

**Table 2:** Laboratory parameters of the study groups.

Variables	Control group (*n* = 40)	Diabetic groups			*P* values
		Normal albuminuria	Microalbuminuria	Macroalbuminuria	
		(*n* = 40)	(*n* = 40)	(*n* = 40)	
*Lipid profile*					
Total cholesterol (mmol/l)	4.8 ± 1.5	4.8 ± 0.7	4.6 ± 0.9	5.1 ± 1.4	0.289
HDL-c (mmol/l)	1.2 ± 0.4	1.2 ± 0.3	1.1 ± 0.4	0.9 ± 1.0	0.196
LDL-c (mmol/l)	6.9 ± 24.5	3.3 ± 0.6	3.4 ± 1.1	1.12 ± 0.4	0.417
Total triglyceride (mmol/l)	1.3 ± 0.8	1.7 ± 0.8	1.4 ± 0.5	2.7 ± 0.9 ^b c^	**<0.001**
*Diabetic assessment*					
FBS (mmol/l)	5.5 ± 5.9	7.9 ± 5.3	12.1 ± 16.7^a^	11.8 ± 4.1^a,b^	**0.004**
HbA1c (%)	4.7 ± 0.4	7.2 ± 0.7^a^	7.5 ± 1.4^a^	9.4 ± 0.8^a,b,c^	**<0.001**
Fasting insulin (mIU/l)	9.6 ± 5.0	25.3 ± 10.4^a^	32.3 ± 14.4^a,b^	37.8 ± 16.8^a,b^	**<0.001**
HOMA-IR index	0.15 ± 0.25	0.8 ± 1.2^a^	0.4 ± 0.3^b^	0.7 ± 0.4^a^	**<0.001**
Total protein (gm/l)	74.2 ± 10.4	73.9 ± 3.2	70.7 ± 4.2	71.9 ± 5.5	0.052
Serum albumin (gm/l)	47.7 ± 7.7	35.0 ± 3.3^a^	34.1 ± 2.4^a^	34.4 ± 2.5^a^	**<0.001**
*Renal function tests*					
Serum urea (mmol/l)	3.5 ± 1.1	4.6 ± 1.0^a^	4.5 ± 0.9^a^	4.5 ± 1.4^a^	**<0.001**
Serum creatinine (*μ*mol/l)	57.7 ± 12.5	56.2 ± 16.0	59.1 ± 9.8	69.2 ± 16.6^a,b,c^	**<0.001**
Albumin/creatinine ratio (*μ*g/mg)	16.7 ± 8.7	10.5 ± 7.8	77.5 ± 65.5	803.5 ± 355^a,b,c^	**<0.001**
eGFR (ml/min/1.73 m^2^)	102.4 ± 17.6	111.2 ± 36.6	107.9 ± 17.2	113.3 ± 22.9	0.232
*Inflammatory markers*					
hs-CRP (mg/l)	0.12 ± 0.08	0.17 ± 0.05^a^	0.17 ± 0.04^a^	0.15 ± 0.02^a,b,c^	**<0.001**
*VDBP analyses*					
sVDBP (*μ*g/ml)	210.3 ± 33.8	202.4 ± 43.9	248.4 ± 36.5^a,b^	299.2 ± 50.6^a,b,c^	**<0.001**
uVDBP/uCr (ng/mg)	127.7 ± 21.9	193.1 ± 141.0	820.4 ± 402.8^a,b^	1458.1 ± 210^a,b,c^	**<0.001**

Data are expressed as mean ± SD. HDL: high-density lipoprotein cholesterol; LDL: low-density lipoprotein cholesterol; FBS: fasting blood sugar; HBA1c: hemoglobin A1c; HOMA: homeostasis model assessment; eGFR: estimated glomerular filtration rate; hs-CRP: high sensitivity C-reactive protein. ^a^Compared to control group. ^b^Compared to diabetic normal albuminuria group. ^c^Compared to diabetic microalbuminuria group. Bold values indicate significance at *P* < 0.05.

**Table 3:** Correlation of uVDBP with the clinical and biochemical features in diabetic nephropathy patients.

Variables	*r*	*P*
*Clinical features*		
Age	−0.088	0.338
Gender	0.009	0.921
BMI	0.115	0.210
FH of DM	0.309	**0.001**
DM duration	0.079	0.394
DR	0.099	0.281
CKD	−0.093	0.311
*Diabetic assessment*		
FBS	0.018	0.849
HbA1c	0.584	**<0.001**
Fasting insulin	−0.155	0.091
HOMA-IR index	−0.099	0.281
*Lipid profile*		
Total cholesterol	0.091	0.324
HDL-c	−0.052	0.575
LDL-c	−0.244	**0.007**
Total triglyceride	0.018	0.849
Total serum protein	−0.202	**0.027**
*Renal function tests*		
Serum urea	0.097	0.294
Serum creatinine	0.010	0.912
Albumin/creatinine ratio	0.775	**<0.001**
eGFR	0.047	0.611
*Inflammatory markers*		
hs-CRP	−0.167	0.089
*Vitamin D-binding protein analysis*		
sVDBP	0.665	**<0.001**

BMI: body mass index; FH of DM: family history of diabetes mellitus; DR: diabetic retinopathy; CKD: chronic kidney; FBS: fasting blood sugar; HOMA: homeostasis model assessment, HDL: high-density lipoprotein cholesterol; LDL: low-density lipoprotein cholesterol; eGFR: estimated glomerular filtration rate; hs-CRP: high-sensitivity C-reactive protein; sVDBP: serum vitamin D-binding protein. Bold values indicate significance at *P* < 0.05.

**Table 4:** Univariate logistic regression analysis.

Variables	Univariate analysis			*P*
	B	OR	(95% CI)	
Duration of DM (year)	−0.013	0.987	(0.947–1.029)	0.544
Fasting blood sugar	0.039	1.040	(0.939–1.151)	0.453
Fasting insulin	0.012	1.012	(0.901–1.136)	0.845
sVDBP	0.027	1.028	(1.003–1.053)	**0.026**
uVDBP/uCr	0.010	1.010	(1.004–1.016)	**0.002**
Total cholesterol	−1.703	0.182	(0.030–1.100)	0.063
Serum creatinine	0.081	1.084	(0.976–1.203)	0.131

sVDBP: serum vitamin D-binding protein; uVDBP/uCr: urinary vitamin D-binding protein/urinary creatinine; B: *β* regression coefficient; OR: odds ratio; CI: confidence interval. Bold values indicate significance at *P* < 0.05.
